# Linked vaccination coverage surveys plus serosurveys among Ethiopian toddlers undertaken three years apart to compare coverage and serologic evidence of protection in districts implementing the RED-QI approach

**DOI:** 10.1016/j.vaccine.2021.08.071

**Published:** 2021-09-24

**Authors:** James D. Campbell, Marcela F. Pasetti, Lisa Oot, Zenaw Adam, Mesfin Tefera, Berhane Beyane, Nigisti Mulholland, Robert Steinglass, Rebecca Krey, Wilbur H. Chen, William C. Blackwelder, Myron M. Levine

**Affiliations:** a Center for Vaccine Development and Global Health, Baltimore, MD 21201, USA; b Department of Pediatrics, University of Maryland School of Medicine, Baltimore, MD 21201, USA; c JSI Research & Training Institute Inc., Arlington, VA, USA; d Ethiopian Public Health Institute (EPHI), Addis Ababa, Ethiopia; e Family & Reproductive Rights Education Program (FARREP), The Royal Women’s Hospital, Parkville, VIC 3052, Australia; f Department of Medicine, University of Maryland School of Medicine, Baltimore, MD 21201, USA; g Department of Epidemiology and Public Health, University of Maryland School of Medicine, Baltimore, MD 21201, USA

**Keywords:** Vaccination, Survey, Measles, Tetanus, Serosurvey, Seroprotection

## Abstract

In low and middle-income countries, estimating the proportion of vaccinated toddlers in a population is important for controlling vaccine-preventable diseases by identifying districts where immunization services need strengthening. Estimates measured before and several years after specific interventions can assess program performance. However, employing different methods to derive vaccination coverage estimates often yield differing results.

**Methods:**

Linked vaccination coverage surveys and seroprotection surveys performed among ~300 toddlers 12–23 months of age in districts (woredas), one per region, of Ethiopia (total, ~900 toddlers) in 2013 to estimate the proportion vaccinated with tetanus toxoid (a proxy for pentavalent vaccine) and measles vaccine. The surveys were followed by implementation of the Reaching Every District using Quality Improvement (RED-QI) approach to strengthen the immunization system.

Linked coverage/serosurveys were repeated in 2016 to assess effects of the interventions on vaccination coverage. Indicators included “documented coverage” (vaccination card and/or health facility register records) and “crude coverage” (documented plus parent/caretaker recall for children without cards). Seroprotection thresholds were IgG-ELISA tetanus antitoxin ≥0.05 IU/ml and plaque reduction neutralization (PRN) measles titers ≥120 mIU/ml.

**Findings:**

Improved markers in 2016 over 2013 include coverage of pentavalent vaccination, vaccination timeliness, and fewer missed opportunities to vaccinate. In parallel, tetanus seroprotection increased in the 3 woredas from 59.6% to 79.1%, 72.9% to 83.7%, and 94.3 to 99.3%. In 2015, the Ethiopian government conducted supplemental measles mass vaccination campaigns in several regions including one that involved a project woreda and the campaign overlapped with the RED-QI intervention timeframe; protective measles PRN titers there rose from 31.0% to 50.0%.

**Interpretation:**

The prevalence of seroprotective titers of tetanus antitoxin (stimulated by tetanus toxoid components within pentavalent vaccine) provides a reliable biomarker to identify children who received pentavalent vaccine. In the three study woredas, the RED-QI intervention appeared to improve immunization service delivery, as documented by enhanced pentavalent vaccine coverage, vaccination timeliness, and fewer missed vaccination opportunities. A measles mass vaccination campaign was followed by a markedly increased prevalence of measles PRN antibodies. Collectively, these observations suggest that wider implementation of RED-QI can strengthen immunization, and periodic linked vaccination surveys/serosurveys can monitor changes.

## Highlights

• A seroprotective tetanus titer indicates a toddler has received pentavalent vaccine.
• Serosurveys document increased seroprevalence post-measles vaccination campaigns.
• Vaccination coverage/serosurveys can assess interventions to improve immunizations.

## 1 Introduction

Immunization services in low- and middle-income countries (LMIC) administer vaccines to children according to national routine immunization schedules that broadly follow guidance from the World Health Organization (WHO) Expanded Program on Immunization (EPI) in each WHO Region and through supplemental immunization activities (SIAs) that include periodic mass vaccination campaigns. Table 1 shows Ethiopia’s EPI schedule. Effective, timely vaccinations reduce the burden of vaccine-preventable diseases. Estimates of vaccination coverage, i.e., the proportion of eligible children who have in fact received a vaccine or vaccine series, aim to provide local, regional, national, and global public health entities with data on the performance of immunization services [1], [2], [3]. Scrutiny of vaccination coverage data can identify performance gaps and pinpoint foci of under-vaccination, which, if improved, can expand vaccination coverage and enhance disease prevention [4], [5]. Identifying districts/neighborhoods where under-vaccinated children reside is of particular importance during the COVID-19 pandemic, as immunization services have deteriorated in many countries [6], [7], [8], [9].

**Table 1:** Recommended vaccinations for Ethiopian children (Source: National EPI Implementation Guideline for Ethiopia, 2020).

Vaccination for Ethiopian infants	
Recommended Age	Vaccines
Birth	BCG-1 OPV-0

6 weeks	Pentavalent-1 PCV-1 OPV-1 Rotavirus-1

10 weeks	Pentavalent-2 PCV-2 OPV-2 Rotavirus-2

14 weeks	Pentavalent-3 PCV-3 OPV-3* (bOPV) IPV-1*

9 months	Measles-1

BCG = Bacillus Calmette-Guérin tuberculosis vaccine;

OPV = Oral polio vaccine

Pentavalent vaccine = Diphtheria toxoid, tetanus toxoid, whole cell pertussis combination vaccine (DPT) combined with hepatitis B vaccine and *Haemophilus influenzae* type b conjugate vaccine;

PCV = Pneumococcal conjugate vaccine

IPV = inactivated polio vaccine; OPV = oral poliovirus vaccine; Pentavalent = DTP-HBV-Hib vaccine; PCV = pneumococcal 10 conjugate vaccine; TT = tetanus toxoid.

After completion of this study (2019), Ethiopia introduced a second dose of measles containing vaccine recommended for 15 months of age.

* New since 2016: Bivalent oral polio vaccine (bOPV) was introduced in April 2016, replacing trivalent OPV (tOPV). IPV was introduced in 2015.

Documentation of vaccination coverage can be estimated by: reported service statistics (“administrative coverage”); review of local healthcare facility vaccination registers and/or family-held individual vaccination cards; parent/caretaker recall; or a combination of these. Many immunization coverage surveys have relied heavily on family-held vaccination records and parent recall [10], [11], [12], [13]. Other surveys have focused on documented vaccination records held by the family (vaccination cards) or by local health facilities (registers) [1], [3]. Nevertheless, both cards and registers may be incomplete or missing [14], and parental/caretaker recall may be inaccurate [3], [13], [15]. Several reviews have addressed the status of Ethiopia’s EPI at different levels [16], [17], [18], [19], [20], [21], [22].

In an individual toddler, serologic protection (seroprotection) against vaccine-preventable diseases, evidenced by antigen-specific serum antibody titers above a recognized protective threshold [23], may emanate from vaccine-derived immunity alone (e.g., tetanus) [3], [24], or from immunity derived either following vaccination or from natural exposure to the wild-type pathogen (e.g., measles) [24]. The proportion of children assumed protected based on vaccination coverage surveys may differ from the proportion with putative protective antibody levels based on serological measures [3]. Measurement of certain specific antibodies provides more objective evidence of individual and population level protection than coverage surveys [3]. However, serosurveys have their own inherent issues including: i) technical challenges of obtaining blood samples from toddlers; ii) processing blood collected in remote, poorly accessible, areas requires special equipment [24]; and iii) correlates of protection may change as new methods become available. Nonetheless, increasing knowledge of population-level protection from vaccine-preventable diseases may help improve vaccination practices and child health.

Since 2003, Ethiopia implemented the WHO/UNICEF “Reaching Every District (RED)” strategy to strengthen RI services, with a focus on bolstering woreda-level (district-level) service delivery [22]. The RED strategy encompasses five operational components: i) planning and management of resources; ii) reaching all eligible populations; iii) engaging communities; iv) supportive supervision; and v) monitoring and use of data for action. Despite implementation of this approach for a decade and putative high vaccination coverage based on administrative estimates (2013 and 2016 WHO-UNICEF [WUENIC] estimates for DTP3 were 72% and 77% respectively) [25], [26], coverage estimates from the 2016 Ethiopian Demographic and Health Surveys (EDHS) indicated low estimates of measles and DTP3 vaccination coverage in Ethiopia overall (53%) and in certain regions, in particular [27], [28]. In 2019 Ethiopia ranked 5th globally with the highest number of unprotected (unvaccinated or undervaccinated) children (1.1 million), and 3rd in Africa behind Nigeria and Democratic Republic of Congo [29].

In 2011, JSI Research & Training Institute, Inc. (JSI), under a Bill & Melinda Gates Foundation (BMGF) learning grant, initiated the Universal Immunization through Improving Family Health Services (UI-FHS) project in Ethiopia [21], [22]. This project explored the dynamics of vaccination and seroprotection and developed recommendations for interventions to achieve and sustain high immunization coverage in Ethiopia, an aspirational goal of the government. To improve RI delivery, JSI applied quality improvement concepts and tools to strengthen implementation of the RED strategy [20], [21], [22]. The “Reaching Every District using Quality Improvement (RED-QI)” approach [30], comprises a package of interventions implemented over 20–24 months to build health worker capacity to improve the management, delivery and utilization of RI services, while harnessing the energy from engaged communities.

**Fig. 1:**
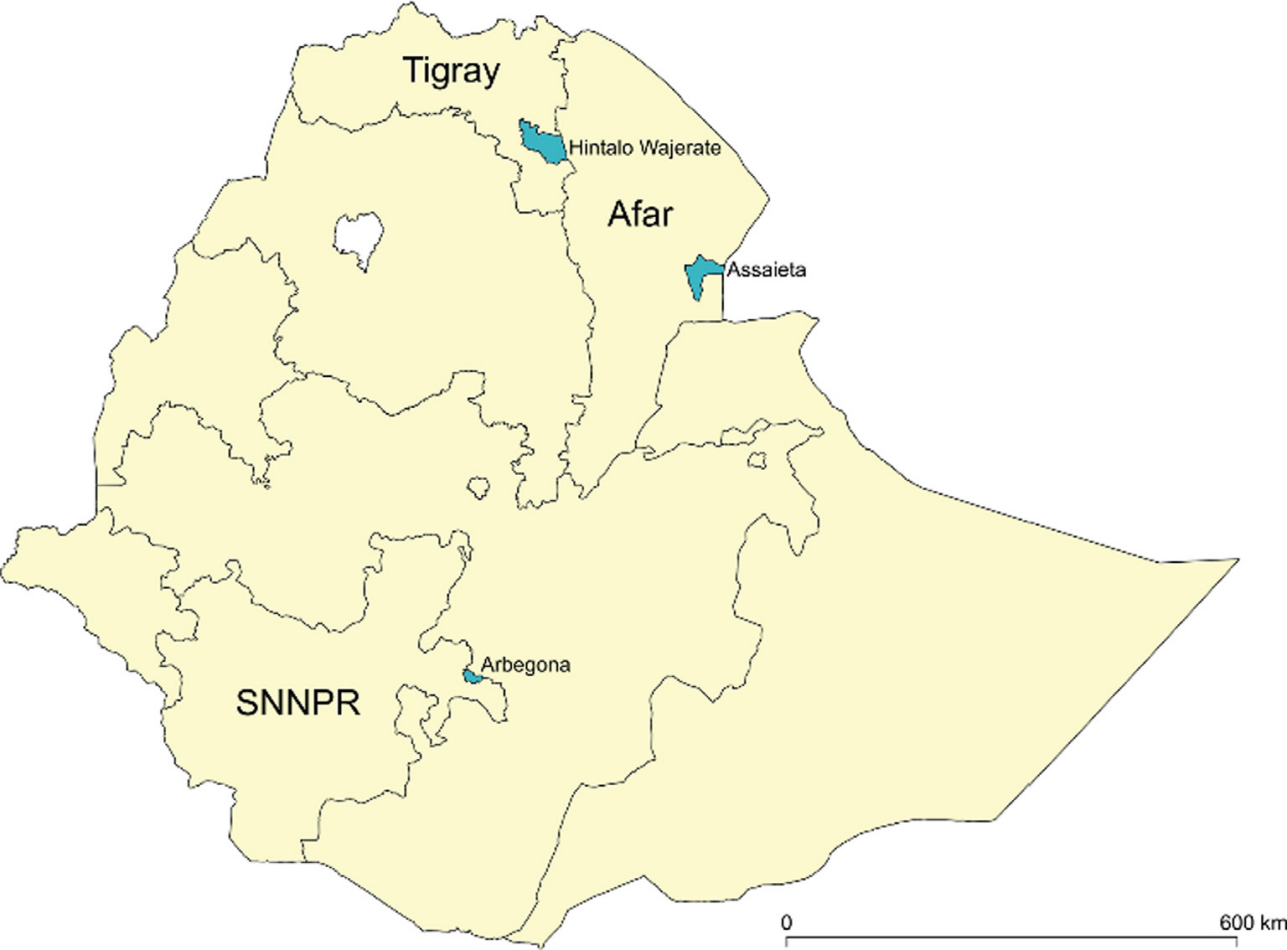
Location of immunization coverage and serologic surveys, Ethiopia, 2013 and 2016. (Figure prepared by Michael Sikorski).

In 2013, JSI and CVD, with BMGF support, undertook immunization coverage surveys in three of the project’s “intervention woredas”, one each located in Afar Region, SNNP (Southern Nations, Nationalities, and Peoples) Region, and Tigray Region (Fig. 1); each vaccination coverage survey was accompanied by a linked serosurvey performed one day later [3], [24]. The 2013 coverage surveys and serosurveys included toddlers 12–23 months of age and infants 6–8 months of age, the latter to assess the timeliness of young infant immunization [3], [24].

A follow-up linked immunization coverage survey/serosurvey was undertaken in these woredas in 2016 (limited to toddlers 12–23 months of age) to assess whether RED-QI demonstrably improved vaccination coverage and seroprotection versus 2013. The 2016 survey was timed to proceed several months after a supplemental mass measles immunization campaign undertaken by the Afar Region government that targeted children 9–59 months of age. Herein we compare results of the 2016 and 2013 linked vaccination coverage surveys/serosurveys to assess the degree of improvement, if any.

## 2 Methods

Ethiopia’s EPI (Table 1) intends for infants to receive first, second, and third doses of pentavalent vaccine at 6, 10, and 14 weeks of age, respectively, followed by a first dose of MCV when the child reaches 9 months of age.

**Pentavalent-1 timeliness:** Infant receives a vaccine on time, at no more than 3 days before 6 weeks but < 12 weeks of age for pentavalent-1.

**MCV timeliness:** Child receives MCV no more than 3 days before 9 months but < 10 months of age.

**Missed opportunity for vaccination:** Any health services contact where a child eligible for vaccination does not receive vaccine.

### 2.1 The RED-QI intervention

The RED-QI comprehensive strategy implemented in the three woredas following the 2013 surveys included training, reinforcement of skills learned through on-the-job support, and peer learning to build health worker capacity to manage and implement key activities that strengthen RI (Table 2). UI-FHS supported health workers to identify communities that needed to strengthen immunization services and to develop immunization microplans to reach underserved populations [30]. Community engagement is critical for RED-QI success. Thus, community members were mobilized to support health workers to identify and solve local problems within the health system, plan immunization services, and implement “defaulter” and “left-out” tracking systems to find and reach unimmunized children. RED-QI also focused on strengthening data quality and use at the health facility level [30], anticipating that better understanding of data would improve data quality, monitoring, and accountability.

**Table 2:** RED Strategy Components, Strategies and Practices.

**RED strategy components**	**RED-QI strategies and practices**
Planning and management of resources, including microplanning	• Developing district, sub-district, and health facility WHO Expanded Programme on Immunization (EPI) microplans. Include community leaders and other stakeholders, such as civil administration, in planning process. • Conducting participatory community mapping to accurately identify catchment populations • Conducting fishbone analyses to identify the root causes of problems • Implementing plan-do-study-act (PDSA) cycles to test solutions crafted by health workers and community members working together

Engaging with communities	• Developing quality work improvement teams (QITs) comprised of health workers and community members to focus on immunization and conduct PDSA cycles, trace defaulters, and obtain community input on immunization program planning, including optimal location and time for vaccination outreach sessions, as well as problem solving • Involving civil administration to elevate issues and mobilize local resources

Conducting supportive supervision	• Increasing focus on health worker capacity building and on-site mentorship, particularly for data analysis and problem solving • Revising existing supportive supervision tools to improve their use for mentoring and on-the-job training

Monitoring and using data for action	• Conducting data quality self-assessment and improving data consistency across standard EPI reporting tools • Building health worker capacity to monitor immunization coverage and drop-out rates to inform health workers’ own actions • Holding quarterly review meetings (QRMs) with both health personnel and local non-health stakeholders to review performance and encourage participants to “think outside the box” to problem solve, mobilize local resources, and flag problems needing national-level attention

Reaching all eligible populations	• Improving capacity of districts and health facilities to plan and implement outreach and mobile services • Using data to identify service needs and to expand the availability of immunization services through static, outreach and mobile sessions • Working to mobilize local resources to overcome barriers to service delivery

In each woreda, RED-QI was introduced through a three-step process with technical support provided over a 13–24 month period, depending on the woreda’s level of health care infrastructure. The first two months of implementation focus on advocacy with key stakeholders and a situational analysis to provide up-to-date information on local context and health system. This is followed by 10–15 months of technical assistance in which QI tools and processes are introduced and the focus is on establishing and strengthening service delivery and management capacity. The final 4–6 months focus on maintaining and sustaining progress in EPI; although sustainability and ownership of the process are critical components throughout implementation, this last step is where collaborative planning for continuation of the approach is central.

RED-QI strategy provides practical methods to support immunization managers and health workers to examine obstacles in the implementation of RED, develop local solutions, and share learning for sustainability and scale-up. The approach enables immunization professionals to take RED from a “what to do” strategy to a “how to” approach for strengthening the routine immunization system. Table 2 summarizes how RED-QI tools and practices operationalize the five components of the RED strategy.

Broad implementation of RED-QI occurred in three phases: 1) *learning phase* - we implemented the approach in the three study woredas; 2) *scaling phase* - we expanded implementation to 100 additional woredas after an intensive process to tailor the approach for weak health system/nomadic contexts, and 3) *institutionalization phase -* we build capacity to implement the approach at regional and zonal levels in Ethiopia. JSI implemented a process of continuous learning and the approach was modified and tailored for each context.

### 2.2 Sites and sample size

**Fig. 2:**
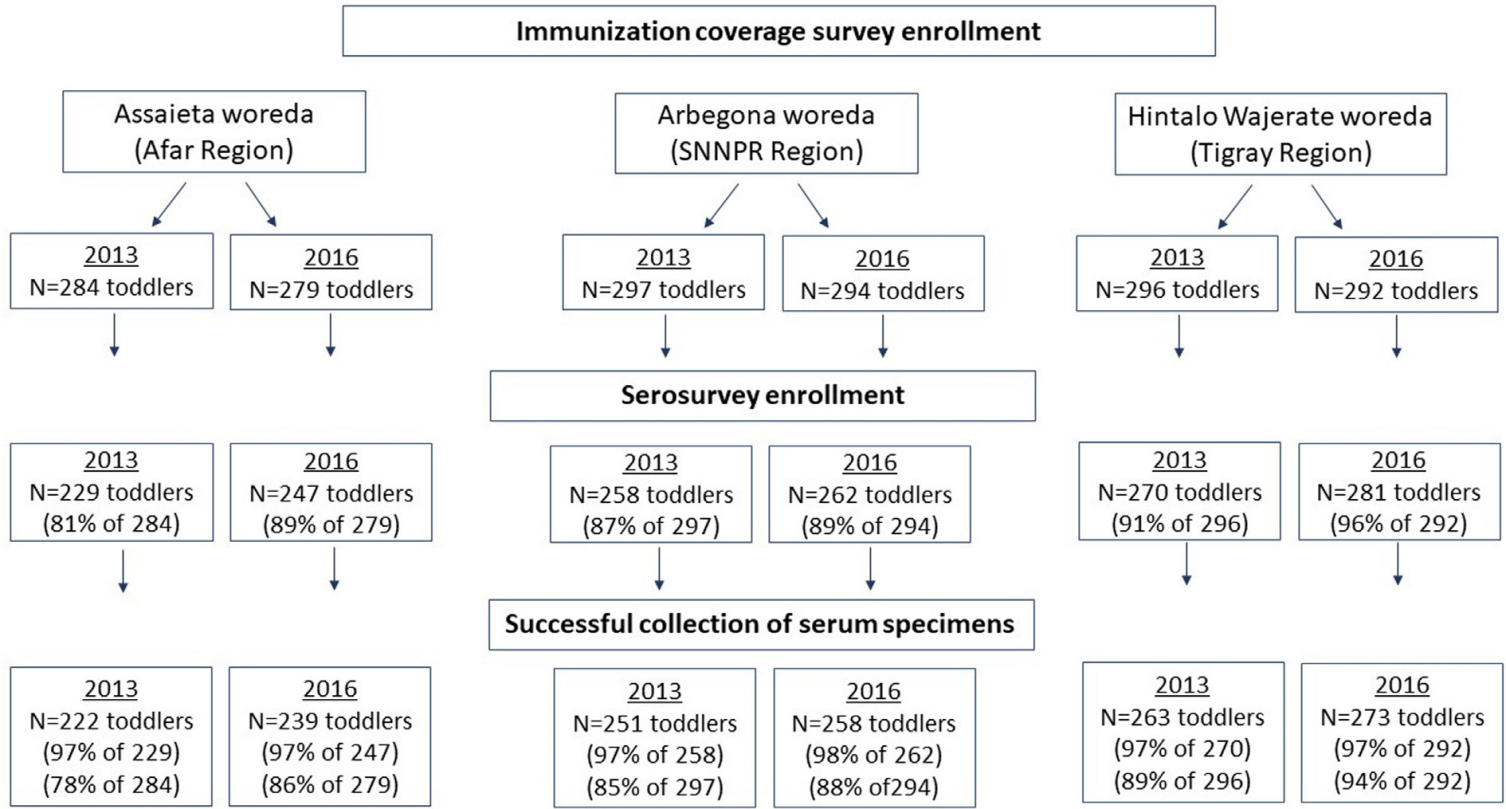
Diagram summarizing the workflow of the vaccination coverage surveys and linked serosurveys performed in 2013 and 2016 in three woredas of Ethiopia. Shown are the number of random households in which age-eligible toddlers were identified, the number of toddlers recruited, and the number from whom a blood specimen was successfully obtained.

The Federal Ministry of Health of Ethiopia (FMOH), in collaboration with JSI, the Center for Vaccine Development and Global Health of the University of Maryland School of Medicine (CVD), and the Ethiopian Public Health Institute (EPHI) performed linked vaccination coverage/serosurveys among toddlers (age 12–23 months) in three woredas: Assaieta (Afar); Arbegona (SNNPR), and Hintalo Wajerate (Tigray). Each woreda survey targeted enrollment of ~300 toddlers (total, ~900) during each survey period, February-April 2013 and February-March 2016 (Fig. 2). FMOH performed a “follow-up” mass immunization with monovalent measles vaccine that targeted children 9–59 months of age in Assaieta from October 2015 through January 2016 and focused on reaching children of nomadic families.

FMOH and JSI selected three woredas in different regions to pilot the RED-QI approach with the woredas reflecting three health system contexts: a very strong health system context (Hintalo Wajerate); a medium health system context in an agrarian area (Arbegona), and a weak health system context in a nomadic area (Assaieta). The survey woredas are relatively close to the regional capital (within 2 h drive) and were selected as “typical” representations for the region.

*Hintalo Wajerate.* Hintalo Wajerate, located in the Debub Misraqawi, SouthEast Zone of Tigray, lies 43 km from the regional capital, Mekelle. Seven health centers (HCs) and 18 health posts (HPs) in 22 kebeles (villages) serve the woreda’s population of 153,505 (2007 census), ~92% of whom live in rural areas [1]. Whereas 14 kebeles are accessible by road throughout the year, the other eight are inaccessible during rainy season (July through August).

*Assaieta.* Assaieta Woreda, located in Zone One of Afar, is 55 km from the regional capital, Semera. Half of Assaieta’s population of 50,803 is pastoralist, moving cattle or camels across the region throughout the year. Assaieta is characterized by extreme climate, with temperatures ranging from 32° to 48 °C, and an average rainfall of only 50 mm/year. This woreda has one hospital, one HC, and 11 HPs that serve 13 kebeles; nine kebeles are accessible year-round by four-wheel-drive vehicles, while four are inaccessible during the rainy season, July-September.

*Arbegona.* Arbegona, located in Sidama Zone, SNPPR, is 77 km southeast of the regional capital, Hawassa. The population of 135,862 is distributed among 39 kebeles served by one hospital, five HCs, and 27 HPs. Arbegona, situated in a mountainous region with few roads, is the least accessible of the three woredas, particularly during rainy season. The woreda health office has only one vehicle, despite the fact that only 12 of 39 kebeles are accessible throughout the year by four-wheel-drive vehicles; 27 kebeles are inaccessible during the long rainy season (May-September).

### 2.3 Coverage surveys

JSI and contractors performed vaccination coverage surveys, following WHO methodology [3], [10], [11], [24]. These surveys, which constitute standard public health practice, obtained information about the proportion of children with a history of having received vaccinations according to the Ethiopian EPI schedule operative from 2012 to 2016, including the number and date of each vaccination dose given [3], [24].

### 2.4 Statistical sampling for clusters and children

To obtain the sample populations, a total of 102 clusters were randomly selected from the most recent list of enumeration areas from the Central Statistics Authority (CSA). The number of infants and children to be surveyed in each enumeration area was then determined on the basis of probability sampling proportionate to size [31]. Finally, a list of households with target-age children was created within the enumeration areas, and households were selected for the survey by systematic random sampling. With an additional 5% adjustment for expected nonresponse, a total of 2080 households (700 each for Arbegona and Hintalo, and 680 for Assaieta) were included. The target census for the coverage survey in each woreda was 300 toddlers 12–23 months of age [3], [24], with typically 9 toddlers selected per cluster. The enrollment target for the serosurvey was 60% of the coverage survey enrollment.

“Documented coverage” is defined as the percent of toddlers age 12–23 months who received 3 doses of pentavalent vaccine with the first given no earlier than 39 days of life as recorded on family-held vaccination cards or in health facility registers (EPI registries) when cards were not available. In addition, parental/caretaker recall on vaccinations received was solicited if a family-held card was unavailable [3]. “Crude coverage” is defined as the percent of toddlers age 12–23 months who received 3 doses of pentavalent vaccine determined by vaccination card, EPI register, or parental/caretaker recall.

### 2.5 Linked serosurveys

Concomitant with the coverage survey in each woreda, CVD and EPHI undertook a serosurvey. The serosurvey protocol and consent procedure were approved by the Ethiopian National Research Ethics Review Committee and the University of Maryland, Baltimore Institutional Review Board. Written informed consent was obtained from parents/caretakers of each child enrolled in the serosurvey. Informed consent was documented by use of a written consent form approved by the IRBs and signed or thumb-printed by the parent or caretaker. Parents of children in the coverage survey were asked to include their children in a seroprotection survey; signed informed consent/permission was received from each parent whose child was included in the serosurvey. Experienced phlebotomists drew blood from each enrolled child, and technicians processed blood specimens on site (Fig. 2) [3], [24].

### 2.6 Serological assays and seroprotection

Sera were tested for tetanus antitoxin as a proxy for receipt of pentavalent vaccine (DTP-Hib-HepB), which contains tetanus toxoid. IgG tetanus antitoxin titers were measured by ELISA using the 1st International Standard for Tetanus Immunoglobulin (NIBSC TE-3) to report IU/mL in test samples [32]. “Seroprotection” for tetanus was defined as a tetanus antitoxin serum IgG titer ≥ 0.05 IU/mL. In the 2013 serosurvey, a titer of ≥ 0.15 IU/mL had been used as the protective cut-off based on previous surveys [32], [33], [34]. However, following the 2013 survey, laboratory studies documented that the CVD ELISA accurately detected tetanus antitoxin in the 0.02–0.10 IU/mL range. It was therefore deemed appropriate to use the 0.05 IU/mL threshold to indicate seroprotection, as this more closely approximates the established minimal protective level (0.01 IU/mL) [35]. A protective tetanus antitoxin level was also considered, by extension, to be a surrogate for adequate immunological responses to the other components of pentavalent vaccine including *Haemophilus influenzae* type b [Hib] conjugate, hepatitis B vaccine, diphtheria toxoid, and pertussis antigens [36], [37], [38].

Measles virus antibodies were measured to assess seroprotection derived either from measles virus containing vaccine (MCV) or from exposure to wild measles virus [39], [40]. Measles plaque reduction neutralization (PRN) assays on sera from a random sample of 100 toddlers from each woreda in 2013 and 2016 were performed [39], [41], using the WHO 3rd International Serum Standard for Anti-Measles (NIBSC 97/648) to report mIU/mL [40]. PRN assays were limited to a sub-sample of only 100 toddlers per woreda to contain costs and expedite testing. Sera from all 300 toddlers per woreda were tested by ELISA for IgG antibodies to a measles virus sonicate antigen [39], [41], an economical assay that correlates with PRN titers [39], [41]. A titer of ≥ 120 mIU/mL was considered the threshold for putative protection against measles [42], whether antibodies were measured by ELISA or PRN.

### 2.7 Statistical analyses

Chi square test (uncorrected) was used to compare proportions. Confidence interval calculation for differences in proportions used normal approximation. A 2-sided p-value ≤ 0.05 was considered statistically significant. No adjustment was made for multiple comparisons.

## 3 Results

### 3.1 Seroprotection

In 2013, 757 of 877 toddlers (86.3%) who participated in the vaccination coverage survey were enrolled in the serosurvey and serum samples were collected from 736 of these 757 (97.2%) [3]. In 2016, 790/865 toddlers (91.3%) who participated in the coverage survey were enrolled in the serosurvey, and serum was obtained from 770/790 (97.5%) (Fig. 2). Surveys were completed in 47 days in 2013 and 41 days in 2016.

**Seroprotection against tetanus.** The percentage of children tested who exhibited protective IgG titers of tetanus antitoxin rose significantly from 2013 to 2016 in all three woredas, with the most prominent increases evident in Assaieta (19.5 percentage points)) and Arbegona (10.8 percentage points), (Table 3). These data indicate that by 2016 objective seroprotection against tetanus was documented in no less than 79.1% of toddlers (Assaieta) and as many as 99.3% of toddlers (Hintalo Wajerate), with Arbegona (83.7%) falling in between. Besides showing that the larger increases in prevalence of protective titers of tetanus antitoxin observed in Assaieta and Arbegona were highly significant, even in a highly-vaccinated population like toddlers in Hintalo Wajerate (94.3% seroprotected in 2013) the tetanus antitoxin biomarker showed that the modest 5 percentage point rise in that woreda was highly significant. Crude coverage survey method showed a parallel highly significant rise in the two woredas that showed large increases in prevalence of seroprotection. In contrast, in highly-immunized Hintalo Wajerate, where the biomarker difference was only 5.0%, crude coverage results between 2013 and 2016 were almost identical (0.5% increase) and non-significant. Documented coverage showed a significant difference only in Hintalo Wajerate and the result was 16% lower coverage in 2016 versus 2013.

**Table 3:** Proportions (%), using various indicators, of toddlers 12–23 months of age in three Ethiopian woredas with evidence of receipt of 3 doses of pentavalent vaccine and proportions with tetanus antitoxin titers ≥ 0.05 IU/mL in 2013 and 2016.

Woreda andindicators to determine seroprotection or coverage*	Proportion (%) seroprotected or covered*		**Difference in percent seroprotected or covered from 2013 to 2016 (95% CI)**	**p-value**
	**2013**	**2016**		
**Assaieta**				
Serosurvey ≥ 0.05 IU/mL	127/213† (59.6%)	189/239 (79.1%)	19.5 (11.1, 27.8)	**<0.0001**
WHO crude survey coverage	75/215 (34.5%)	110/239 (46.0%)	11.1 (2.2, 20.1)	**0.0159**
Documented coverage‡	57/215 (26.5%)	68/239 (28.5%)	1.9 (-6.3, 10.2)	0.6440

**Arbegona**				
Serosurvey ≥ 0.05 IU/mL	183/251 (72.9%)	216/258 (83.7%)	10.8 (3.7, 17.9)	**0.0030**
WHO crude survey coverage	103/251 (41%)	147/258 (57%)	15.9 (7.4, 24.5)	**0.0003**
Documented coverage‡	91/251 (36.3%)	74/258 (28.7%)	-7.6 (-15.7, 0.5)	0.0680

**Hintalo Wajerate**				
Serosurvey ≥ 0.05 IU/mL	248/263 (94.3%)	271/273 (99.3%)	5.0 (2.0, 8.0)	**0.0010**
WHO crude survey coverage	229/263 (87.1%)	239/273 (87.6%)	0.5 (-5.2, 6.1)	0.8692
Documented coverage‡	217/263 (82.5%)	181/273 (66.3%)	-16.2 (-3.5, -9.0)	**<0.0001**

**All 3 woredas**				
Serosurvey ≥ 0.05 IU/mL	558/727 (76.8%)	676/770 (87.8%)	11.0 (7.2, 14.9)	**<0.0001**
WHO crude survey coverage	407/729 (55.8%)	496/770 (64.4%)	8.6 (3.6, 13.6)	**0.0007**
Documented coverage‡	365/729 (50.0%)	323/770 (41.9%)	-8.1 (-13.2, -3.1)	**0.0017**

† n/N (%)

‡ Documented information on whether a child received vaccine came from family-held vaccination cards and from EPI vaccine registries at health facilities, culminating in the measurement of “documented coverage”. “WHO survey coverage” includes documented coverage on vaccinations plus parent/caretaker recall, if a vaccination card was not available; recall information was not considered “documented”.

* A child was “covered” for pentavalent vaccine if at the time of the survey according to document or recall, she/he had received 3 doses of pentavalent vaccine, with the first dose having been given no more than 3 days before 6 weeks of age.

**Seroprotection against measles.** We tested random sample subsets of sera from 100 of the 300 toddlers in each woreda by PRN. In each woreda the proportion of toddlers having a protective PRN titer ≥ 120 mIU/mL rose modestly in 2016 over the seroprotective PRN titers evident in 2013 (Table 4). Nevertheless, in 2016 only 36.0% (Arbegona), 50.0% (Assaieta), and 76.0% (Hintalo Wajerate) of toddlers exhibited PRN titers ≥ 120 mIU/ml. Since some persons with PRN titers < 120 mIU/ml are also protected [43], we also analyzed the percent of children in each woreda who would be considered seroprotected if the PRN cut-off was ≥ 80 mIU/mL (Table 4). This lower cut-off modestly raised the estimated prevalence of seroprotection in 2016 by 8 percentage points in Assaieta and Hintalo Wajerate and by 7 percentage points in Arbegona. Even using the 80 mIU/mL cutoff, the prevalence of seroprotection in the woredas still only reached 43% (Arbegona), 58% (Assaieta) and 84% (Hintalo Wajerate) (Table 4), far below the ~90–95% prevalence of protection sought by FMOH to interrupt sustained transmission of measles.

**Table 4:** The proportion of toddlers age 12–23 months in three Ethiopian woredas in 2013 and 2016 with evidence of seroprotective titers of anti-measles virus antibodies measured by two different survey methods (plaque reduction neutralization [PRN] and IgG-ELISA) are shown using a cut-off titer of ≥ 120 mIU/mL as seroprotective. The prevalence of PRN titers ≥ 80 mIU/mL are also shown. (Note – in areas where measles disease remains endemic, anti-measles antibodies may derive from vaccine or from infection with wild type measles virus).

Woreda and indicators to determine seroprotection or coverage*	Proportion (%) seroprotected or covered*		**Difference in percent seroprotected or covered 2016 vs 2013 (95%CI)**	**p-value**
	**2013**	**2016**		
**Assaieta**				
Serosurvey- PRN 120 mIU/mL	31/100 (31.0%)	50/100 (50.0%)	19.0 (5.7, 32.3)	**0.0093**
Serosurvey- PRN 80 mIU/mL	33/100 (33.0%)	58/100 (58.0%)	25.0 (11.6, 38.4)	**0.0006**
Serosurvey- ELISA	76/215 (35.4%)	131/239 (54.8%)	19.5 (10.5, 28.4)	**<0.0001**
WHO crude survey coverage	46/215 (21.4%)	164/239 (68.6%)	47.2 (39.2, 55.3)	**<0.0001**
Documented coverage‡	35/215 (16.3%)	70/239 (29.3%)	13.0 (5.4, 20.6)	**0.0012**

**Arbegona**				
Serosurvey- PRN 120 mIU/mL	26/100 (26.0%)	36/100 (36.0%)	10.0 (-2.7, 22.7)	0.13
Serosurvey- PRN 80 mIU/mL	29/100 (29.0%)	43/100 (43.0%)	14.0 (0.8, 27.2)	**0.039**
Serosurvey- ELISA	53/251 (21.1%)	55/258 (21.3%)	0.2 (-6.9, 7.3)	0.96
WHO crude survey coverage	106/251 (42.2%)	169/258 (65.5%)	23.3 (14.8, 31.7)	**<0.0001**
Documented coverage‡	61/251 (24.3%)	57/258 (22.1%)	-2.2 (-9.5, 5.1)	0.55

**Hintalo Wajerate**				
Serosurvey- PRN 120 mIU/mL	63/100 (63.0%)	76/100 (76.0%)	13.0 (0.4, 25.6)	**0.046**
Serosurvey- PRN 80 mIU/mL	71/100 (71.0%)	84/100 (84.0%)	13.0 (1.6, 24.4)	**0.028**
Serosurvey- ELISA	172/263 (65.4%)	116/273 (42.5%)	-22.9 (-31.1, -14.7)	**<0.0001**
WHO crude survey coverage	194/263 (73.8%)	216/273 (79.1%)	5.4 (-1.8, 12.5)	0.14
Documented coverage‡	175/263 (66.5%)	150/273 (55.0%)	-11.6 (-19.8, -3.4)	**0.006**

**All woredas**				
Serosurvey- PRN 120 mIU/mL	120/300 (40.0%)	162/300 (54.0%)	14.0 (6.1, 21.9)	**0.0006**
Serosurvey- PRN 80 mIU/mL	133/300 (44.3%)	185/300 (61.7%)	17.3 (9.5, 25.2)	**<0.0001**
Serosurvey- ELISA	301/729 (41.3%)	302/770 (39.2%)	-2.1 (-7.0, 2.9)	0.41
WHO crude survey coverage	346/729 (47.5%)	549/770 (71.3%)	23.8 (19.0, 28.7)	**<0.0001**
Documented coverage‡	271/729 (37.2%)	277/770 (36.0%)	-1.2 (-6.1, 3.7)	0.63

The prevalence of measles seroprotection measured by IgG-ELISA using a cut-off of ≥ 120 mIU/mL generally paralleled the seroprotection values measured by PRN in Assaieta but not so in Arbegona or Hintalo Wajerate. One consistent observation, irrespective of the immunologic assay or titer cut-off applied, was the increase in measles seroprotection in Assaieta in 2016 over 2013; rises of 19.0 percentage points (PRN ≥ 120 IU/ml), 25.0 percentage points (PRN ≥ 80 IU/ml) and 19.5 percentage points (ELISA ≥ 120 IU/ml) were recorded by the three serological measurements (Table 4).

### 3.2 Coverage surveys

In Table 3, Table 4, estimates from “documented coverage” and “crude coverage” survey indicators are compared to seroprotection data. Evidence for receipt of pentavalent vaccine among toddlers in the three woredas in 2016 versus 2013, as estimated by the two coverage indicators, is summarized in Table 3. In each woreda, the “crude coverage” method, which incorporates the widest sources of data, including parental/caretaker recall, provided higher overall estimates of vaccination. Even the “crude coverage” indicator method markedly under-estimated the seroprotection levels recorded in all three woredas.

Based on the 2016 coverage survey, documented coverage estimates for receipt of MCV-1 were only 22.1%, 29.3%, and 54.9%, respectively, for Arbegona, Assaieta, and Hintalo Wajerate (Table 4). “Crude coverage” indicators provided higher estimates of MCV1 coverage in 2016 of 65.5%, 68.6%, and 79.1%, respectively, for Arbegona, Assaieta, and Hintalo Wajerate.

In the 2016 survey, 70% of caregivers overall (60.2% in Arbegona, 68.8% in Assaieta, 81.2% in Hintalo Wajerate) reported having received a vaccination card, while 30% denied ever receiving a vaccination card (Table 5). Among caregivers who reported having received vaccination cards, a sizable proportion (50%) had either lost their card or were unable to show it at the time of the survey (Table 5). As the overall percentage of caregivers able to produce a vaccination card at the time of the coverage survey was low (20.1% in Arbegona, 35.5% in Assaieta, 49.3% in Hintalo Wajerate), UI-FHS visited health facilities and reviewed health care registers to confirm parental recall of vaccination for children without vaccination cards. Even with additional documentation from health facilities, only 55.8% of toddlers overall included in the 2016 survey had documented evidence of any vaccination. In the 2013 vaccination coverage survey, information on whether a vaccination card was seen was obtained only during household visits, and only 261/877 (30%) of households showed a card.

**Table 5:** Availability of home-based records in children 12–23 months by time of survey, 2016.

**Source of data**	**Number of households with toddlers enrolled**	**N (%) that reported having received an immunization card**	**N (%) of households that showed a card on the day of the survey**	N (%) unable to produce an immunization card*	**N (%) that reported never having received a card**
Arbegona	294	177 (60.2)	59 (20.1)	118 (40.1)	117 (39.8)
Assaieta	279	192 (68.8)	99 (35.5)	93 (33.3)	87 (31.2)
Hintalo Wajerate	292	237 (81.2)	144 (49.3)	93 (31.8)	55 (18.9)

* Not able to produce card = (% who ever received − % observed on day of survey) / % who ever received a card.

### 3.3 Timeliness of vaccination

**Fig. 3:**
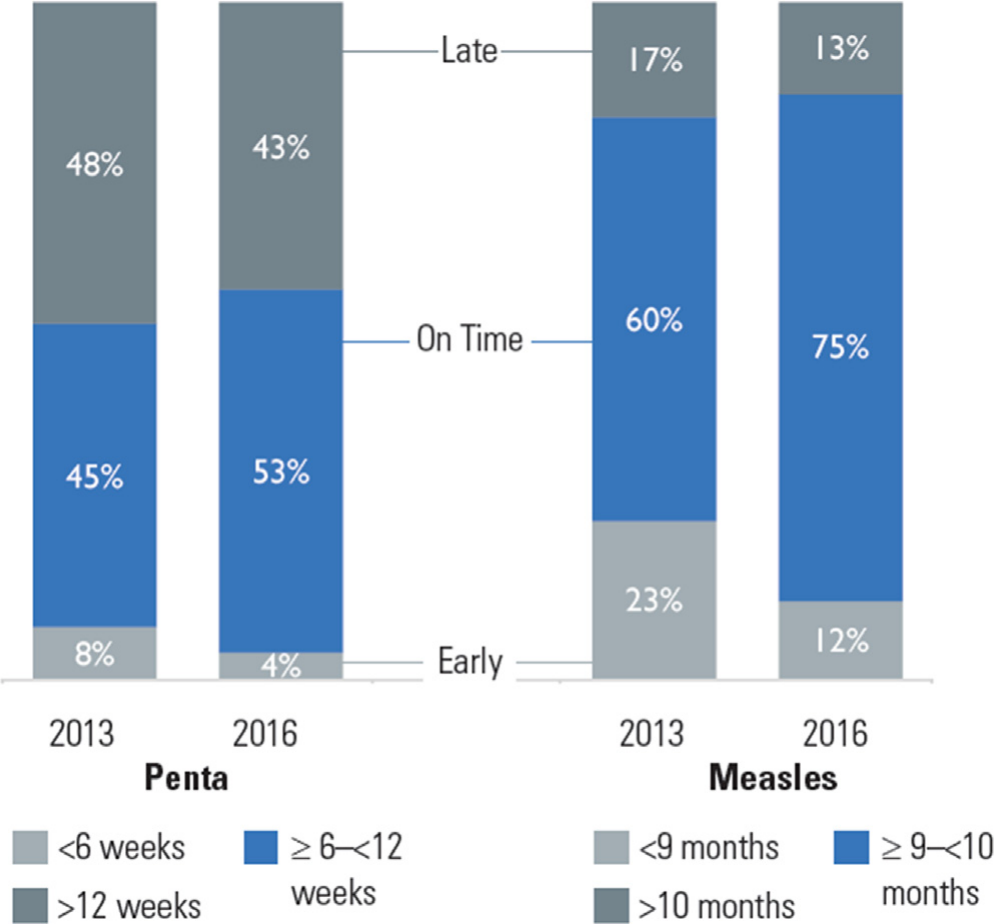
Comparison of timeliness of the first dose of pentavalent 1 vaccine (penta1) and of measles containing vaccine (MCV1) among children aged 12–23 months with documented (card and register) evidence of vaccination in the three survey woredas.

Ethiopia’s EPI intends for infants to receive first, second, and third doses of pentavalent vaccine at 6, 10, and 14 weeks of age, followed by MCV1 at 9 months of age (Table 1). The percentage of children receiving a vaccine on time, at no more than 3 days before 6 weeks but < 12 weeks of age for pentavalent-1 and between no more than 3 days before 9 months but < 10 months for measles, improved for both pentavalent-1 (8 percentage points) and measles (15 percentage points) vaccination between 2013 and 2016 (Fig. 3). However, many children continue to be vaccinated after the recommended schedule, particularly for pentavalent-1 (43% in 2016), leaving children at prolonged risk. Despite improvements from 2013, in 2016 4% of children were still being vaccinated with penta1 and 12% with MCV before the recommended age, potentially resulting in sub-optimal immune responses (Fig. 3, Fig. 4A, Fig. 4B).

**Fig. 4A:**
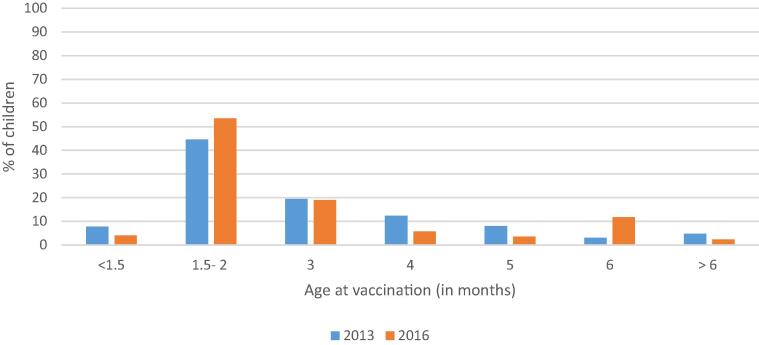
Comparison of timeliness of penta1 vaccination among children aged 12–23 months with documented (card and register) evidence of vaccination in all three woredas, 2013 (n = 422) and 2016 (N = 247).

**Fig. 4B:**
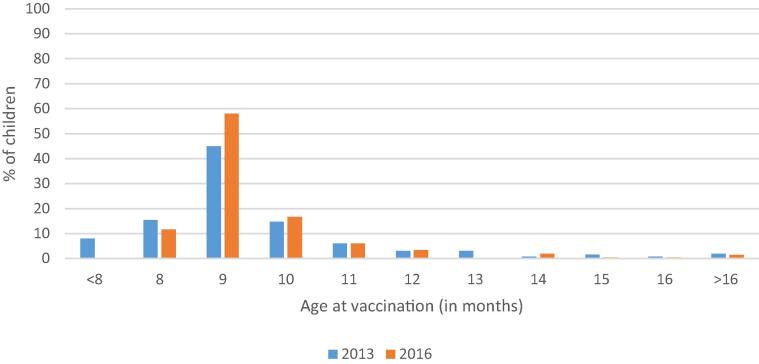
Comparison of timeliness of 1st dose measles vaccination among children aged 12–23 months with documented (card and register) evidence of vaccination in all three woredas, 2013 (n = 265) and 2016 (n = 215).

### 3.4 Missed opportunities for vaccination

In 2013, 94/877 surveyed children (10.7%) received late doses of pentavalent vaccine at ≥ 9 months of age, i.e., when they were also eligible to receive MCV; these 94 included 48 (51%) from Arbegona, 32 (34%) from Hintalo Wajerate (34%), and 14 (15%) from Assaieta. Among these 94 children, 55 (58.5%) failed to receive concomitant measles vaccine when they got their delayed pentavalent dose, thus documenting many missed opportunities to vaccinate against measles. Of these 55 children who didn’t receive MCV, 31 (56%) were from Arbegona, 12 (22%) from Assaieta, and 12 (22%) from Hintalo Wajerate (22%). In the 2016 survey, the number of children receiving delayed pentavalent vaccine at ≥ 9 months of age fell from 94 to only 30, a 68% drop. Of these 30 children (15 from Arbegona [50%], 5 from Assaieta [17%], and 10 from Hintalo Wajerate [33%]), only 5 of 30 (16.7%) failed to receive concomitant measles vaccination; 3 of the 5 were from Arbegona (60%), 2 from Assaieta (40%), and none from Hintalo Wajerate. Thus, the frequency of late pentavalent vaccinations among children who were old enough to receive MCV1 dropped by two-thirds from 2013 to 2016, and the proportion of those who did not receive concomitant MCV also fell from (58.5% [55/94] to 16.7% [5/30]), providing further evidence of improved immunization services. While the absolute number of children who showed up late for a pentavalent vaccine dose fell substantially, the proportion coming from each woreda in 2013 and 2016 was almost identical: Arbegona (51%, 50%); Assaieta (32%, 33%); Hintalo Wajerate (15%, 17%). This may indicate that RED-QI was having a comparable impact in all three woredas.

Further evidence of the improvement of timeliness of vaccination by 2016 compared to 2013 derives from the median age among those who received MCV1. In 2013 the median age of measles-vaccinated toddlers was 417 days (~14 months) of age, whereas in 2016 it declined to 365 days (12 months), approaching the targeted age of 9–10 months.

## 4 Discussion

Immunization against specific vaccine-preventable diseases diminishes the morbid and fatal ravages of these infections in poorly resourced countries [44], [45]. However, daunting logistical impediments must be overcome to supply vaccines and assure that they have been properly maintained in all steps of the cold chain so that even in remote areas of low-income countries, infant/toddler vaccinations will reliably confer immunity [46]. Assessing the quality of immunization services is challenging. Health Ministries rely in part on monthly administrative data reports to track progress in vaccinating target populations in districts and states. However, in many countries, including Ethiopia, administrative data are notoriously inaccurate [2]. Thus, Health Ministries seek alternative and complementary methods to quantify immunization coverage and protection. Over the decades, one approach has been to perform vaccination coverage surveys [4]. Advocates and critics have provided lucid perspectives on the advantages and limitations of types of coverage surveys [1], [15], [47]. Some argue for a more technology-based approach that links serosurveys to coverage surveys [3], [24], [48], or to perform serosurveys alone [23], to determine seroprotection based on antibody measurements to specific vaccine antigens. Useful clinical specimens include whole blood, serum, eluates of dried blood spots from filter paper, and oral fluid.

The vaccination coverage surveys/linked serosurveys that we undertook in Ethiopia in 2013 and 2016 have generated insights that enrich knowledge in this field. These linked surveys carried out three years apart indicate that the RED-QI approach, instituted after the first survey to strengthen RI at the local level, likely did achieve significant improvements.

The value of tetanus antitoxin measurement as a biomarker to assess whether a child has received pentavalent vaccine has been demonstrated in our surveys. A strength of this bioassay is that in a toddler (an age by which placentally-transferred antibodies have disappeared) antitoxin derives only from vaccination with tetanus toxoid. A limitation is that the antitoxin titer cannot differentiate whether the child has received one, two, or three doses of pentavalent vaccine. Besides the tetanus toxoid component of DTP vaccine within pentavalent vaccine, the most common Hib conjugate encountered in pentavalent combinations utilizes tetanus toxoid as the carrier protein. So, infants nowadays who receive pentavalent vaccine are injected with more tetanus toxoid than in years past when they received only DTP. A tetanus antitoxin titer ≥ 0.05 IU/ml indicates that the child has received at least one dose of pentavalent vaccine.

Another relevant finding was that following the measles mass vaccination campaign in late 2015 in Assaieta, the crude vaccination coverage survey method, which includes parental/caretaker recall, appeared to be more reliable than documented coverage estimates alone that exclude parent/caretaker recall. One plausible explanation is that the unusual situation of a parenteral vaccine being delivered via mass immunization by mobile units to a wider age group (often at the domicile level, as among transient nomadic encampments in Afar) and with heightened social mobilization and political support, is an imprinting event that parents subsequently recall.

Our results corroborate concerns over using a cut-off of ≥ 120 mIU/ml of PRN antibodies as a biomarker of vaccine-derived protection against measles [49], and of the overall utility of this antibody, since there is currently no way to differentiate vaccine-derived from infection-derived PRN antibodies. Despite the limitations of measles PRN antibody as a biomarker for evidence of measles vaccination in an individual child in areas where wild measles virus is circulating, its use has been advocated in low- and middle-income countries to detect an improvement in community-wide seroprotection after MCV mass immunization campaigns, by performing pre- and post-campaign surveys [23]. Our results in Assaieta support this general experience, as the prevalence of putative protective titers of both PRN and IgG-ELISA anti-measles virus sonicate antibodies rose markedly in 2016, following the mass measles vaccination campaign of late 2015, and compared to 2013.

Vaccination coverage surveys can still play a key role at the woreda level where, for example, detailed analysis of documented vaccination data can identify the timeliness of administration of specific vaccines, which is integral to their effectiveness. Improvements in levels of coverage and protection from 2013 to 2016 for both measles and tetanus provide evidence that the RED-QI approach, which builds capacity of health management and health staff to identify, plan, and implement robust vaccination services, improved the reach and quality of immunization services in the surveyed woredas. Improvements in the timeliness of vaccinations from 2013 to 2016 and an increase in the percentage of children who received their first dose of pentavalent vaccine and measles vaccine on time further demonstrate improvements in delivery of RI services. RED-QI builds the capacity of health workers to identify and solve problems using local data. In each woreda, health workers indicated that infants often start but do not complete the full course of vaccinations. Health staff worked to address this problem through establishment of locally-tailored defaulter tracking systems and engagement of the community in identification and tracking of pregnant women and newborns. We conclude that these activities improved coverage and timeliness of vaccination in the woredas. UI-FHS also focuses on reducing missed opportunities for vaccination and the endorsement of FMOH policy to open a vial of vaccine for even a single eligible child. This advocacy and support may have contributed to the decrease in missed opportunities for measles vaccination in 2016 over 2013.

In Assaieta, documented coverage increased from 2013 to 2016 for both tetanus and measles, while it decreased between 2013 and 2016 in Arbegona and Hintalo Wajerate for both tetanus and measles. One possible explanation for the inconsistency is that as the quality of data improves, documented coverage may decrease as coverage becomes more accurate. Since RED-QI focuses specifically on improving the quality and consistency of data at the point of its generation, improved data quality may have impacted documented coverage. Thus, the lower documented coverage in 2016 in two woredas may be accounted for by inflated coverage estimates in the first survey (2013) and more accurate, albeit lower, coverage estimates in the follow-on survey in 2016, which occurred after systematic attempts to improve the quality of record keeping. Second, issuance of vaccination cards to caregivers and vaccination registrations at health facilities continues to be highly irregular and incomplete, in part due to the periodic unavailability of documentation materials (cards, family folders). This may also have contributed to poor documentation processes and practices which were beyond what JSI and the RED-QI can address. Unreliable supplies of cards is a well-recognized problem globally [14].

In Ethiopia there is a need to reach more children with measles vaccine and to ensure that when vaccinated, they receive potent vaccine, delivered optimally. Despite notable progress in each woreda, survey findings indicate a need to further strengthen elements of the RI system. Review of the measles data suggests issues related to handling of MCV that should be investigated. Survey findings in Arbegona, for example, indicate that children are getting injected/vaccinated but are not seroconverting (attaining putatively protective titers of measles antibodies). Faulty cold chain management of MCV and mistakes in techniques of mixing MCV with diluent during vaccination sessions may be contributing to this situation in Arbegona. Without investments in strengthening the quality of vaccination for measles, and in coverage for both MCV1 and MCV2, recurring outbreaks of measles in Ethiopia will continue [50]. Ensuring that children are vaccinated and protected is of even greater importance as the FMOH implements strategies to identify and reach children with missed vaccinations because of RI service disruptions due to the COVID-19 pandemic [8]. An approach that strengthens the RI system at the local level can benefit needy districts elsewhere in Africa and Asia; a challenge is to achieve the benefits at a sustainable cost [51]. Others working in Africa have corroborated the benefits of joint coverage and serosurveys, to better understand levels of protection in the community, including after mass vaccination campaigns [48].

Our study has limitations. For one, where we review pooled coverage data from the three woredas, we mask the uniqueness of each individual woreda and the woreda-specific obstacles faced by health workers. Yet the heterogeneous microcosm of just the three woredas that we studied reflects challenges faced by health authorities at the national and sub-national levels as they strive to monitor immunization services and select effective and sustainable solutions to strengthen RI [2]. Another limitation is that we designed the 2013 vaccination coverage survey using methods recommended by the 2005 version WHO cluster survey manual [10], current at the time. Since then, methods have been modified to address potential biases inherent within the 2005 methodology that may influence accuracy and reliability of vaccination coverage estimates. Reliance on parental recall, poor documentation of vaccination performance, and low availability of vaccination cards affect the accuracy and validity of measurements [52].

Another limitation is that with survey data from only three of 830 woredas of Ethiopia we cannot extrapolate widely the applicability of improvements we attribute to RED-QI. On the other hand, data from the woredas in which we worked may be fairly applicable to other woredas in the respective regions. A fundamental limitation is that our surveys cannot account for all potential confounders (human, financial, logistical, infrastructural) that could have in parallel led to changes over the study period. A contrasting limitation is that with our coverage surveys performed only three years apart, this may be too short to measure adequately the improvements that RED-QI may be achieving within the intervention woredas [52].

Finally, even if measles antibody is measured using the PRN assay, measles serology has limitations as a biomarker, including: i) only limited data provide evidence for selecting the cut-off of ≥ 120 mIU/ml as indicating a protected individual [42]; ii) neither the PRN assay, nor ELISA, can differentiate whether the antibodies derive from infection with wild type measles virus or from vaccination with measles containing vaccine.

## 5 Conclusions

We have confirmed that linking vaccination coverage surveys with seroprotection surveys involving the same children is feasible and that successive vaccination coverage surveys/linked serosurveys performed several years apart can constitute a useful strategy to assess the performance of both RI services and mass measles immunization campaigns. We found that the prevalence of tetanus antitoxin at a protective cut-off of ≥ 0.05 IU/ml to be a reliable biomarker to assess receipt of pentavalent vaccine through RI services, while measles antibody measurements remain useful to assess the increased prevalence of seroprotected children consequent to mass measles immunization campaigns, if pre-and post-campaign antibody prevalences can be compared. Serological methods enhance what can be learned from vaccination coverage surveys alone; performing repeat surveys in the same districts several years apart can provide valuable insights into program performance over time.

## Funding

A grant (OPP1017350) from the Bill and Melinda Gates Foundation was awarded to JSI Research and Training Institute (JSI) (Dr. Adam Zenaw, Principal Investigator), which issued a sub-contract to the Center for Vaccine Development of the University of Maryland School of Medicine (CVD) (Prof. M. M. Levine, Principal Investigator), Baltimore, MD. In turn, CVD issued a sub-subcontract to the Ethiopian Public Health Institute (EPHI), Addis Ababa, Ethiopia (Mrs. Berhane Beyene, Principal Investigator).

## CRediT authorship contribution statement

**James D. Campbell:** Methodology, Formal analysis, Writing – review & editing. **Marcela F. Pasetti:** Methodology, Formal analysis, Writing - Review & editing. **Lisa Oot:** Methodology, Data curation, Formal analysis, Supervision, Writing – review & editing. **Zenaw Adam:** Methodology, Data curation, Writing – review & editing. **Mesfin Tefera:** Data curation, Writing – review & editing. **Berhane Beyane:** Data curation, Writing – review & editing. **Nigisti Mulholland:** Data curation. **Robert Steinglass:** Methodology, Writing – review & editing. **Rebecca Krey:** Writing – original draft. **Wilbur H. Chen:** Data curation, Formal analysis, Validation. **William C. Blackwelder:** Data curation, Formal analysis, Validation. **Myron M. Levine:** Methodology, Formal analysis, Writing – original draft, Writing – review & editing, Supervision.

## Declaration of Competing Interest

The authors declare that they have no known competing financial interests or personal relationships that could have appeared to influence the work reported in this paper.

## Appendix A Supplementary material

All authors attest they meet the ICMJE criteria for authorship.
